# Author Correction: Task-dependent representations of stimulus and choice in mouse parietal cortex

**DOI:** 10.1038/s41467-019-08368-x

**Published:** 2019-01-18

**Authors:** Gerald N. Pho, Michael J. Goard, Jonathan Woodson, Benjamin Crawford, Mriganka Sur

**Affiliations:** 1 Department of Brain and Cognitive Sciences, Massachusetts Institute of Technology, Cambridge, MA 02139 USA; 2 Picower Institute for Learning and Memory, Massachusetts Institute of Technology, Cambridge, MA 02139 USA; 3 Department of Organismic and Evolutionary Biology, Harvard University, Cambridge, MA 02138 USA; 4 Department of Molecular, Cellular, and Developmental Biology, University of California, Santa Barbara, Santa Barbara, CA 93106 USA; 5 Department of Psychological & Brain Sciences, University of California, Santa Barbara, Santa Barbara, CA 93106 USA

Correction to: *Nature Communications;* 10.1038/s41467-018-05012-y; published online 03 July 2018

In the original version of this Article, the Acknowledgements section was inadvertently omitted. This has now been corrected in both the PDF and HTML versions of the Article.

