# Supplementary material for: Task-dependent representations of stimulus and choice in mouse parietal cortex
Source: Nat Commun. 2018 Jul 3;9:2596. doi: 10.1038/s41467-018-05012-y (PMC6030204; doi:10.1038/s41467-018-05012-y)
Supplement: Supplementary file 1 — Supplementary Information [file 41467_2018_5012_MOESM1_ESM.pdf]

Supplementary Information

Task-dependent representations of stimulus and choice in mouse parietal cortex

Pho et al.

6 Supplementary Figures

1 Supplementary Table

Supplementary Figures

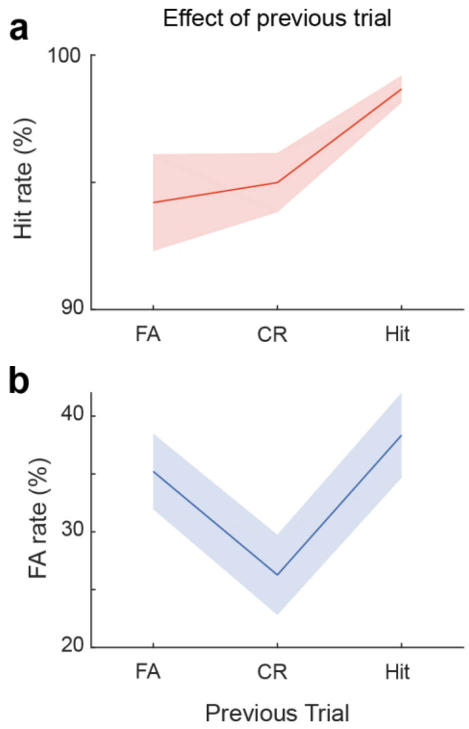

Supplementary Figure 1. Consumption of quinine does not reduce lick rate on subsequent trials.

Hit rate (**a**) and False Alarm (FA) rate (**b**) following different trial types (FA, CR or Correct Reject, Hit). Shading indicates SEM across all imaging sessions (n = 40). If quinine consumption (FA trials) discouraged licking on subsequent trials, one would expect a reduction of lick rate following FA trials compared to CR trials. Instead, well-trained mice showed similar Hit rates following FA versus CR trials (p = 0.26, Wilcoxon signed-rank test) and showed slightly higher False Alarm rates following FA versus CR trials (p = 0.13).

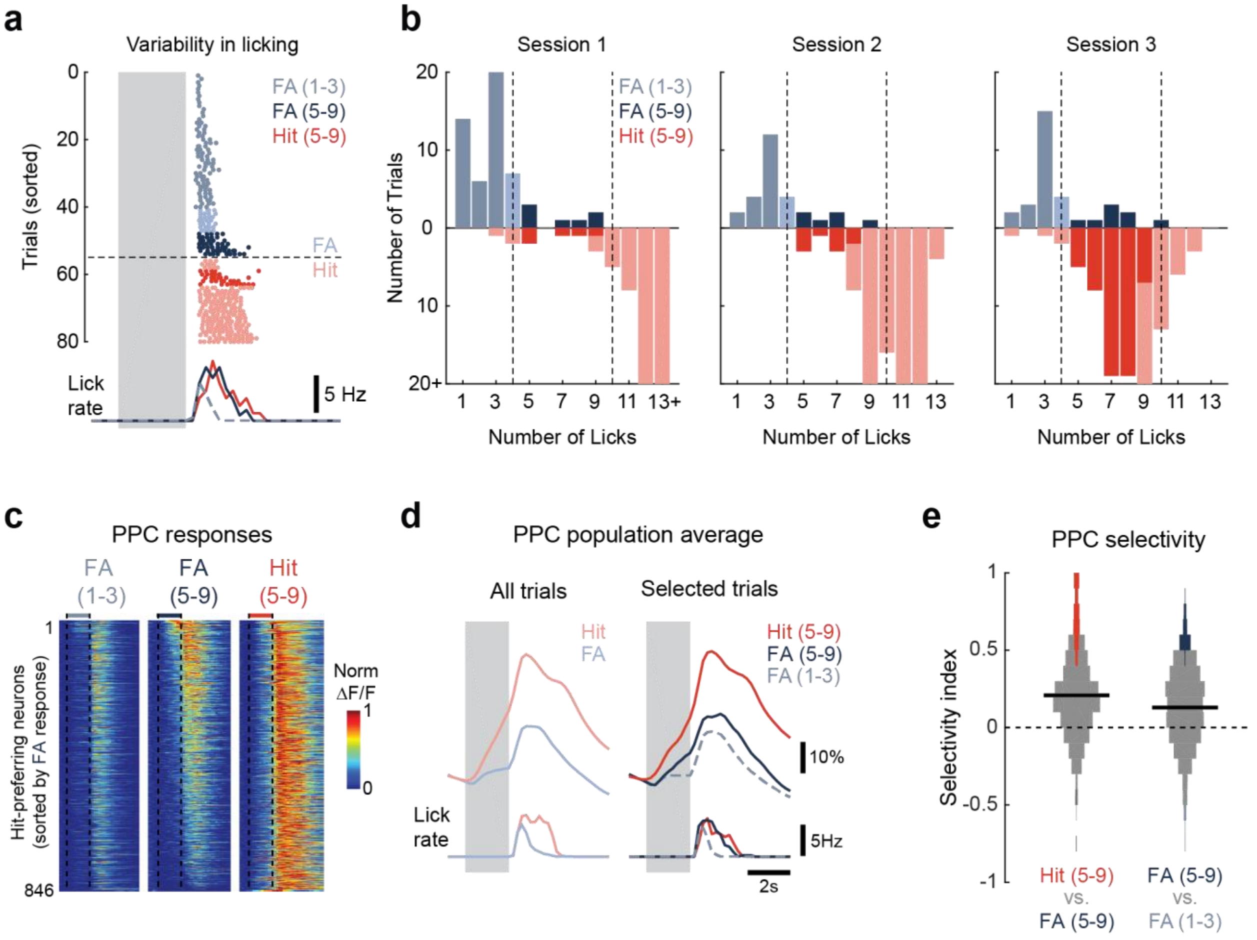

Supplementary Figure 2. PPC responses depend on both motor output and trial type. Some mice exhibited significant variability in licking behavior across False Alarm (FA) trials. For these mice, trials with different number of licks were separated to evaluate the sensitivity of PPC responses to motor output and trial type. (**a**) An example session, Session 1 in (b), with variability in licking behavior across FA trials. Top, trials were separated into FA (blue) and Hit (red), and then sorted by number of licks (not all Hit trials are shown). FA trials with 1-3 licks or 5-9 licks and selected Hit trials (5-9 licks) are highlighted with darker colors. Bottom, average lick rate for selected trials. (**b**) Histograms of lick number from FA (blue) and Hit (red) trials in three sessions (two mice). FA trials with 1-3 licks or 5-9 licks were selected for further analysis. Hit trials with 5-9 licks were randomly selected to match the average number of licks in the FA (5-9) condition. (**c**) Trial-averaged responses of Hit-preferring PPC neurons (n = 846 across 3 sessions). Responses

for each neuron are normalized by peak response across the three conditions. Neurons are sorted by descending strength of FA (5-9 licks) response. Vertical dashed lines demarcate duration of stimulus. (**d**) Top, population trial-averaged PPC responses during all Hit and FA trials (left), and during selected Hit and FA trials (right). Average lick rate (bottom). (**e**) Violin plots (rotated histograms) of selectivity index for PPC neurons, computed using stimulus-period responses. Two comparisons were made for each neuron. Horizontal lines indicate mean, and colored bars indicate neurons with significant individual modulation ($p < 0.05$).

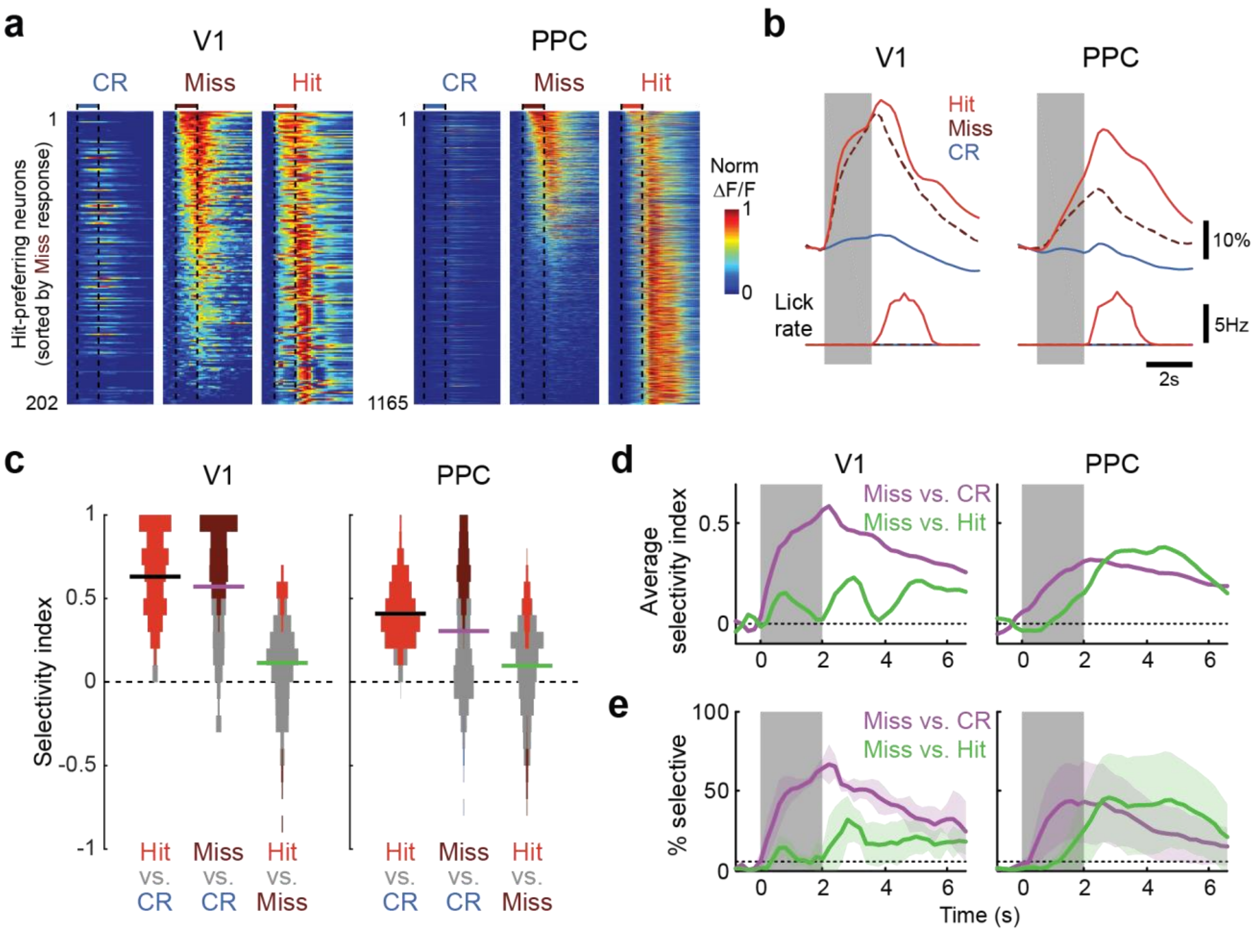

Supplementary Figure 3. PPC responses on Miss trials are variable.

Only Hit-preferring neurons were used in all panels of this figure. (**a**) Trial-averaged responses during Miss trials, for recordings with at least five Miss trials, in V1 (left; 202 neurons across 3 sessions) and in PPC (right; 1165 neurons across 8 sessions). Responses for each neuron are normalized by peak response across Correct Reject (CR, left, blue), Miss (center, dark red), and Hit trials (right, red). Neurons are sorted by descending strength of Miss response. Vertical dashed lines demarcate duration of stimulus. (**b**) Population trial-averaged responses (top) during Hit, Miss (dashed), and CR trials, for V1 (left) and PPC (right). Average lick rate (bottom). (**c**) Violin plots (rotated histograms) of selectivity index for V1 (left) and PPC (right) neurons, computed using stimulus-period responses. Three comparisons were made for each neuron in each area (Hit > CR, Miss > CR, Hit > Miss, see **Supplementary Table 1**). Horizontal lines

indicate mean, and colored bars indicate neurons with significant individual modulation ($p < 0.05$). (**d**) Average selectivity between trial types, computed separately at each time-point. (**e**) Percentage of neurons with significant ($p < 0.05$) positive selectivity at each time-point. Shading indicates mean ± SEM across sessions. Dotted line indicates the expected percentage by chance.

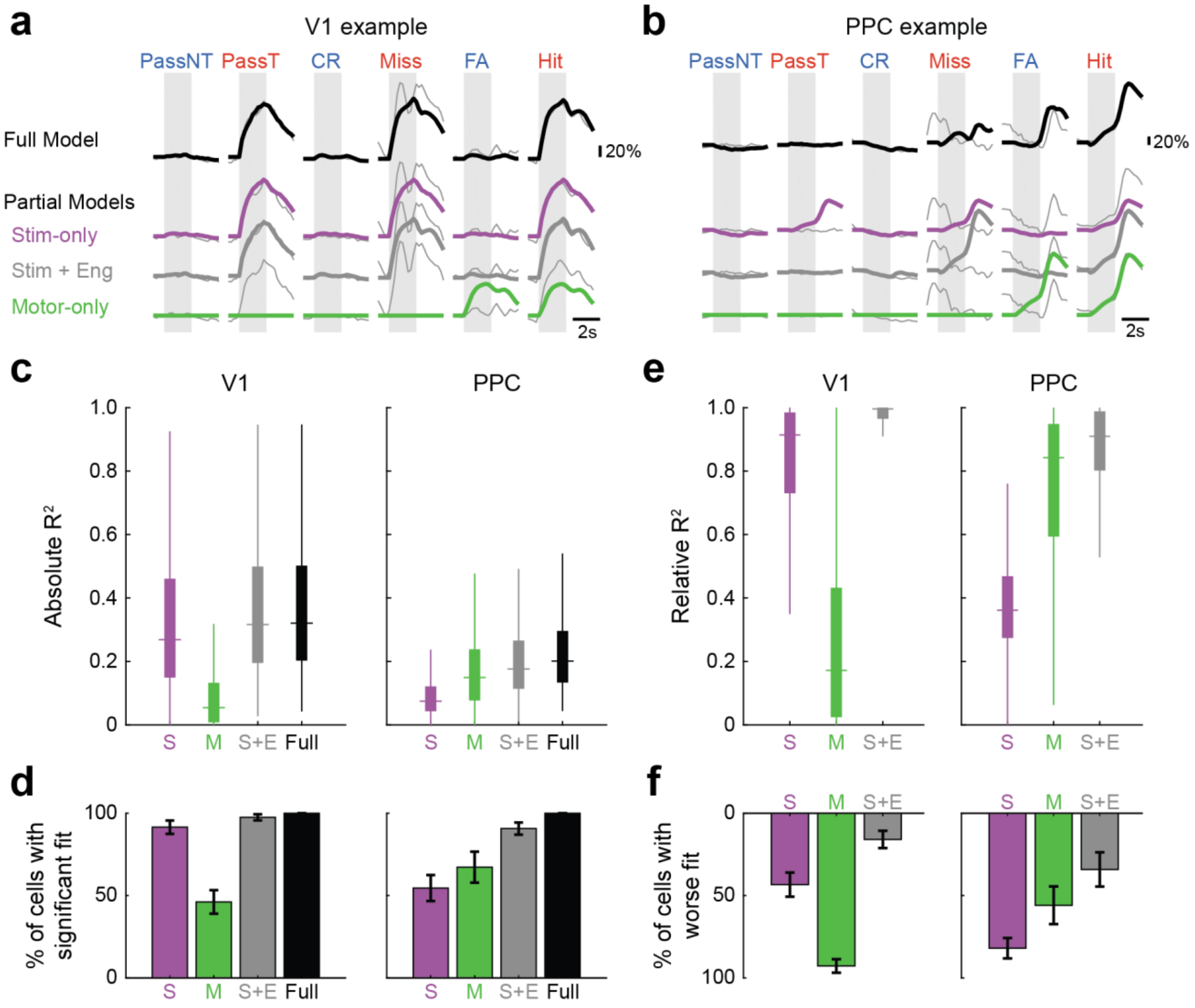

Supplementary Figure 4. Partial GLM models are insufficient to explain PPC responses.

Three partial GLM models were trained for each V1 and PPC neuron: a stimulus-only model (Stim-only, purple), a stimulus plus engagement model (Stim + Eng, gray), and a motor-only model (green). The same training and test sets were used across models. (**a**) Performance of the full model (black) and the partial models (colored) for an example V1 neuron (same cell as Fig. 4a). Predictions are plotted with thick lines, and trial-averaged test data is plotted with thin lines across Passive (PassNT, PassT), Engaged No-Lick (Miss, FA), and Engaged Lick (FA, Hit) conditions. Shaded region represents time of stimulus presentation. Performance of the Stim-only (R^2 = 0.80) and Stim+Eng (R^2 = 0.81) were comparable to that of the Full model (R^2 = 0.81),

whereas the Motor-only model performed poorly ($R^2 = 0.20$). (**b**) Same as (a) but for a PPC neuron (same as Fig. 4d). Performance of the Motor-only ($R^2 = 0.54$) and Stim+Eng ($R^2 = 0.55$) were comparable to that of the Full model ($R^2 = 0.58$), whereas the Stim-only model performed poorly ($R^2 = 0.23$). (**c**) Boxplots (with center line as median, box as interquartile range, and whiskers as 1.5 * IQR) of model performance in absolute R^2 for all four models. Only neurons with significant fits ($p < 0.05$) using the Full model are included (V1: 1674 neurons, PPC: 2573 neurons). (**d**) Percentage of neurons with significant fits ($p < 0.05$) using the partial models. Error bars represent bootstrapped SEM across sessions. (**e**) Boxplots of relative R^2 for all three partial models. (**f**) Percentage of neurons with significantly worse fit ($p < 0.05$) using the partial model relative to the full model.

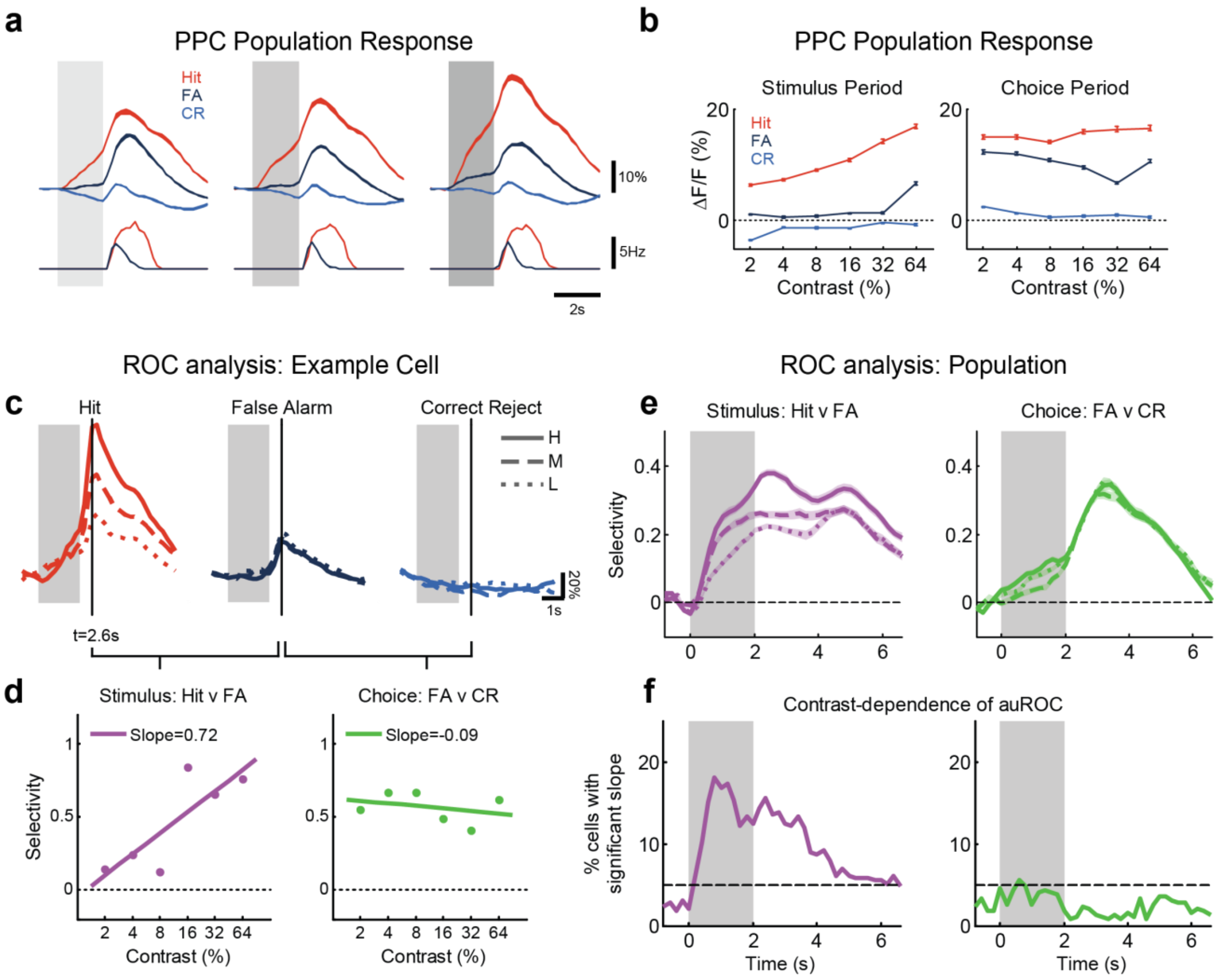

Supplementary Figure 5. PPC encodes both contrast-dependent sensory signals and contrast-independent choice signals.

(**a**) Population average of PPC responses across contrasts during Hit, False Alarm (FA), and Correct Reject (CR) trials (top). Responses are averaged across low (2 or 4%, left), medium (8 or 16%, middle), and high (32 or 64%, right) contrast. Average lick rate (bottom). Light gray shaded regions demarcate duration of stimulus. (**b**) Time-averaged population response as a function of contrast during the Stimulus period (left, 0 to 2 s) or during the Choice period (right, 2 to 3.5 s) for Hit, FA, and CR trials. Choice period responses were measured relative to preceding Stimulus period response. (**c**) Response of an example PPC neuron during Hit trials (left), False Alarm trials (middle), and Correct Reject trials (right), across different contrasts, from low (L,

dotted) to medium (M, dashed), to high (H, solid). (**d**) False Alarm (FA) trials were compared with Hit trials to assess Stimulus selectivity (left), and with CR trials to assess Choice selectivity (right) at a single time-point but across multiple contrasts. Best-fit line as a function of log contrast is plotted along with its slope (arbitrary units). Stimulus selectivity is contrast-dependent, as seen with the significant positive slope, whereas choice selectivity is not. (**e**) Average stimulus selectivity (left) and choice selectivity (right) across all PPC neurons as a function of time and of contrast, from low (L, dotted) to medium (M, dashed), to high (H, solid). Shading indicates SEM. (**f**) Fraction of PPC neurons with significant contrast-dependence in stimulus selectivity (left) or choice selectivity (right), as measured by slope of auROC with respect to log contrast. Dashed line indicates fraction expected by chance.

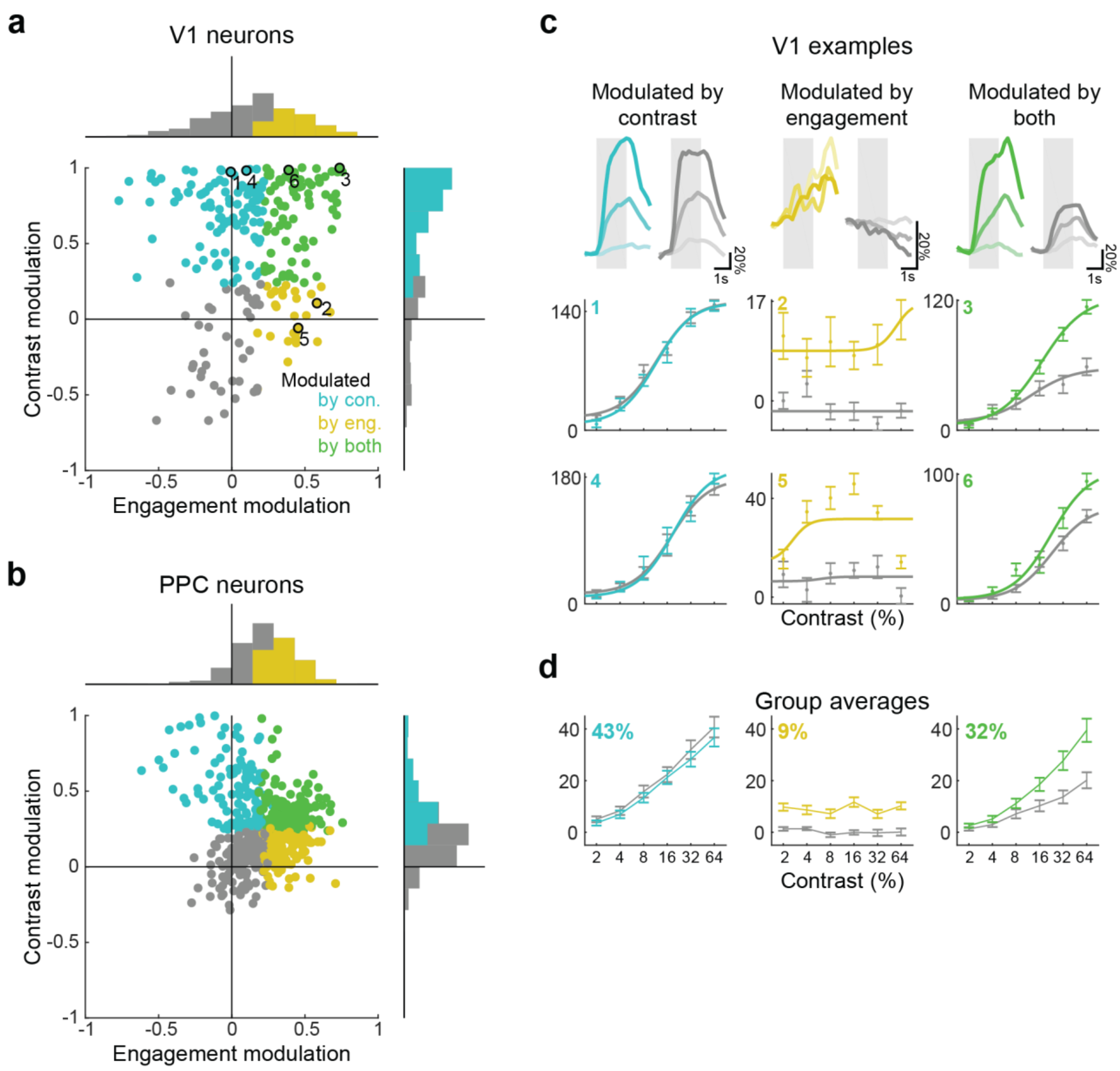

Supplementary Figure 6. V1 neurons are mostly modulated by contrast but also show some heterogeneity.

(**a**) Scatter plot and histograms of contrast modulation versus engagement modulation for all target-preferring V1 neurons (n = 250) imaged during the variable contrast task. Colored bars in histograms (same as Fig. 5e, g) indicate neurons with significantly positive modulation by contrast (cyan) or engagement (yellow). Colored dots on scatter plot demarcate neurons with significantly positive modulation by contrast alone (cyan), engagement alone (yellow), or both contrast and

engagement (green). Individual examples in (c) are marked with the corresponding number. (**b**) Same as (a), but for PPC. Data is the same as in Fig. 6b but re-plotted with same axes as in (a). (**c**) Trial-averaged responses (top row) and contrast-response functions (middle and bottom rows) of example V1 neurons that were significantly modulated by contrast (left column), engagement (middle column), or both (right column). Modulation index values for each example can be found by referring to (a). (**d**) Group-averaged contrast-response functions. Percentages indicates proportion of V1 neurons within each group.

Supplementary Table

Fig	Comparison	Stimulus	Licking	Interpretation
2h	Hit vs. CR	Different	Different	Cannot distinguish stimulus vs choice-related signals
3	FA vs. CR	Same	Different	Isolate choice-related signals
3	Hit vs. FA	Different	Different # of licks	Cannot distinguish stimulus vs choice-related signals
S1	Hit (5-9 licks) vs. FA (5-9 licks)	Different	Same	Isolate stimulus-specific signals (but decision/preparation process may still differ)
S1	FA (5-9 licks) vs. FA (1-3 licks)	Same	Different	Isolate choice-related signals
S2	Miss vs. CR	Different	Same	Isolate stimulus-specific signals
S2	Hit vs. Miss	Same	Different	Isolate choice-related signals

Supplementary Table 1. Error trial comparisons and their interpretation. We define “choice-related” selectivity as any motor preparatory- or decision-related signal. For most comparisons, any selectivity measured after the stimulus period may include signals related to reward, punishment, or motor output. We therefore restricted analyses of selectivity to the stimulus period.
